# Measurement System for Lossy Capacitive Sensors: Application to Edible Oils Quality Assessment

**DOI:** 10.3390/s19194299

**Published:** 2019-10-04

**Authors:** Ahmed Fendri, Ahmed Yahia Kallel, Hanen Nouri, Hamadi Ghariani, Olfa Kanoun

**Affiliations:** 1 Technische Universität Chemnitz, 09111 Chemnitz, Germany; ahmedfendri@yahoo.com (A.F.); ahmed-yahia.kallel@s2017.tu-chemnitz.de (A.Y.K.); hanen.nouri@etit.tu-chemnitz.de (H.N.); 2 National Engineering School of Sfax, University of Sfax, Sfax 3038, Tunisia; hamadi.ghariani@enis.rnu.tn

**Keywords:** edible oils, dielectric measurement, relative permittivity, dielectric losses, capacitive sensor, total polar compounds, acidity, capacitance, resistance

## Abstract

This paper aimed to develop a portable, low-cost, and easy-to-use measurement system for oil quality degradation assessment. The main two chemical parameters affected by frying are the total polar compounds (TPC) and free fatty acids. The system should characterize the change of chemical parameters by measuring the changes in its dielectric parameters. The dielectric parameters, relative permittivity, and conductivity are measured by measuring the capacitance and resistance of a capacitive sensor dipped in oil. The main challenges are that the corresponding changes of the capacitance and resistance are very small and the presence of stray effects. For this reason, the measurement system should be able to detect changes in capacitance and resistance with high resolution and with good immunity to stray effects. The proposed measurement system is based on the conversion of impedance to voltage and time and combining, therefore, having two measurement methods in one circuit. In this way, it is possible to measure the dielectric and resistive parameters and not only the relative permittivity as was done in previous works. The results showed a strong correlation between the chemical and electrical parameters with a coefficient of determination in the range of 0.9.

## 1. Introduction

Edible oils are used on a daily basis in culinary, mainly in frying. Unacknowledged, the oil used in frying could be reused for economic reasons. This practice has proven to be hazardous due to quality degradation caused by the chemical reactions induced in the heating process. These chemical reactions result in the formation and population of the total polar compounds and free fatty acids. These components cause serious illnesses, e.g., heart diseases [1,2]. The refrying process could issue human-perceivable changes such as the change in flavor, quality, and texture change [1]. While each oil is subject to a different change for human perceptibly, the chemical reactions remain similar. For this reason, objective tests are often favored. Moreover, the aforementioned newly-born components are influenced by the speed of the chemical reactions. Present regulations have set a threshold value of total polar components (TPC) of 25%, greater than which the oil must be disposed of [3]. Several methods have been deployed for TPC measurements, among which is the column chromatography. However, this technique is complicated, slow, and requires many chemical catalysts, several of which are carcinogenic.

Several techniques were developed to simplify and accelerate the measurement process, such as absorption spectroscopy and gas and liquid chromatography. These alternative techniques are much simpler but are limited to the laboratory and require the use of expensive devices [4,5,6,7,8,9]. GC is also used. Yet, as it is relatively complex, time-consuming, and requires high-cost devices making it unpopular for oil quality measurement. Some of the applications of GC include the measure of fatty acid composition and the detection of volatile components such as sterols and triglycerides [10,11]. LC is similar to the GC technique. It is usually used to find the amount of a chemical component within a mixture of different components [12,13]. Dielectric spectroscopy is another simple, quick, and non-destructive technique which provides information about the dielectric properties of a medium exposed to an electric field. The dielectric parameters, relative permittivity, and dielectric losses depend on the composition of the medium, reflecting its ability to store energy (relative permittivity) and to dissipate energy (dielectric losses). The DS technique is widely used to determine the type of edible oil, the concentration of each component, to detect adulteration, to measure the water content, and to detect the degree of deterioration of fried oil [14,15,16,17,18]. The measurement setup consists usually of a capacitive sensor connected to a RLC (Resistor Inductance Capacitor) meter or an impedance analyzer. In the work done in [14] and in [16], it is found that the relative permittivity of different edible oils is independent of the frequency for frequencies lower than 1 MHz. The main challenges for this measurement are the stray effects caused by the stray capacitances and the dielectric losses presented as a parallel resistance. To achieve a higher measurement accuracy, the stray effects must be minimized or taken into account in the measurement system. The value of the stray capacitances is usually in the same or higher range as the measured capacitance (pF range) and are not constant. The Dielectric losses, also defined as conductance losses, exist in all capacitive sensors and vary from some MΩ to GΩ depending on the sensor geometry and the characteristic of the medium under investigation.

Several solutions are available for the measurement of the value of the capacitive sensor. However, most of these solutions are proven to deviate in relation to the stray capacitances and conductance losses as they assume that the sensor is purely capacitive. The oscillation circuits are among the simplest circuits for capacitance measurement. They rely on extracting the information of the relaxation time which is in direct proportion to the capacitance. Among oscillation-based measurement techniques are the relaxation-based technique [19] and the voltage controlled techniques [20,21,22,23]. The main disadvantage of these circuits is the high deviation due to the presence of the conductance losses. The switched capacitance circuits are considered more immune to conductance losses and stray capacitances but the implementation of such circuits is difficult since they need many control signals and/or a voltage-controlled current source. Unless they are directly implemented on chip, the measurement errors will be high [24,25]. Other circuits in literature are sigma-delta modulation [26,27], dual slope [28,29], and phase shift circuit. The latter can provide an accurate measurement within a very limited measurement range [30,31,32]. Circuits that rely on voltage measruements as I/Q modulation [33,34] and bridge circuits can provide a stable and accurate measurement [35,36,37,38]. While the I/Q modulation technique’s implementation requires a phase-shifter which is sensitive to phase mismatches, the bridge is proven to be more stable [35,36,37,38].

In this work, a system combination of the capacitive sensor for oil quality assessment together with a measurement circuit based on the combination of the bridge and phase detector techniques is investigated. The capacitive sensor is conceived to be able to sense a small variation in the dielectric parameters, while being relatively small and is immersed into the oil to be investigated. On the other hand, the proposed interface circuit measures the change of the dielectric parameters of the oil, through the capacitive and resistive values, while minimizing the effect of the stray components.

## 2. Reference Measurements

In order to evaluate and calibrate the proposed measurement system, chemical and dielectric reference measurements were done. The chemical measurement consists of the measurement of acidity and total polar components by the means of the titration technique and the Testo 270. The dielectric measurement is realized using a laboratory measurement system based on an impedance analyzer and capacitive sensor.

### 2.1. Chemical Measurement

Five different oils were purchased from the local market for this investigation. The oils were selected from ordinary edible oils: One sunflower, one corn, one rapeseed, and two olive oils. 1.5 L of each type of oil was placed in a laboratory heater and heated at 220 °C. The frying process was carried out at 5 h intervals for 30 h, continuously. After each 5 h interval of heating, a sample of 200 mL was taken, cooled to room temperature in a vacuum desiccator to avoid oxidation with the ambient air. At the end of the experiment, a total of 28 samples were prepared where each type of oil had 7 samples: Fresh, 5 h, 10 h, 15 h, 20 h, 25 h, and 30 h fry time.

**Figure 1:**
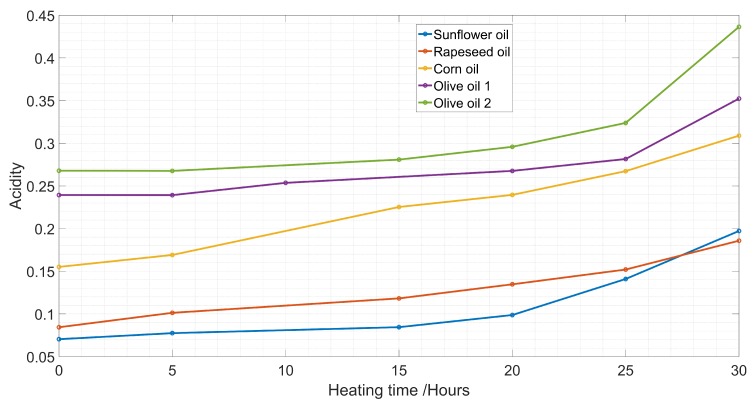
Increase of acidity with heating hours.

The measurement of acidity was done according to the standard ISO 660 based on the titration technique. The acidity increased with the increase of heating time with a pronounced result starting after 25 h of frying (Figure 1). This result was in agreement with the result found in previous works [39,40]. Olive oil had more unsaturated fatty acids than sunflower, rapeseed, and corn oils. This explains why olive oil samples had the highest values of acidity. Indeed, short and unsaturated fatty acids are more soluble in water than long and saturated fatty acids [41], which facilitates hydrolysis and oxidation during frying, and hence the acidity is higher [42].

The content of total polar compounds was determined using the measurement system Testo 270 with an accuracy of ±2%. The device’s tip was dipped in oil for at least 15 s until the TPC percentage and temperature stabilized [43,44].

**Figure 2:**
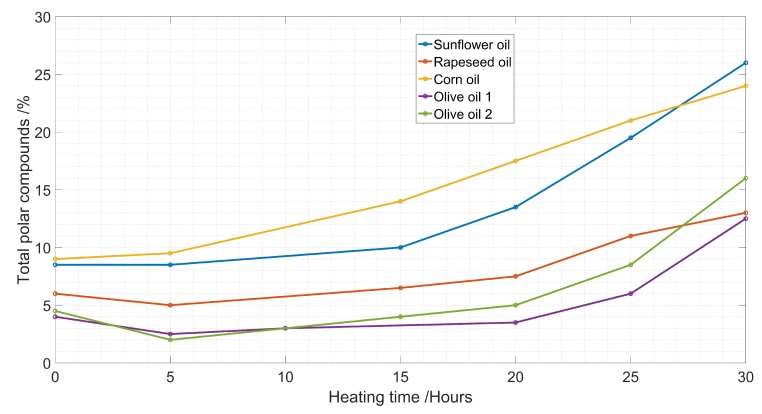
Increase of total polar compounds with heating hours.

In the early stage of heating (less than 15 h), the TPC had only a slight change. This was because the oil was not hot enough to react with oxygen and moisture (Figure 2). Afterwards the TPC increased with the sunflower oil, reaching a TPC of 26 % at 30 h. Olive oil and rapeseed oils proved to be more monotonously exponentially increasing. The TPC of rapeseed oil also increased, but with a different slope compared to the others.

### 2.2. Dielectric Measurement

**Figure 3:**
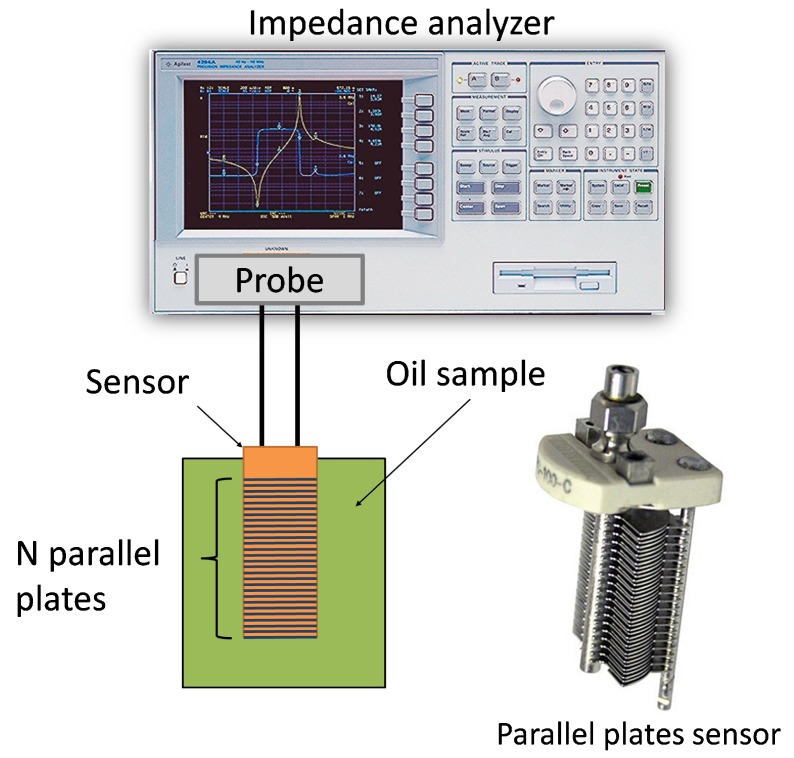
Measurement set-up for reference measurement including the parallel plates’ sensor.

The dielectric measurement was done with the help of a parallel plates sensor connected to an impedance analyzer (Figure 3). The used sensor was composed of 20 fixed and 21 mobile armatures. The maximum capacitance value was obtained when all armatures were fully overlapped. This sensor was used since it could provide a high capacitance value and high resolution to the change of the dielectric parameters.

The capacitance of the sensor in the air was $C_{0}$ = 136.7 ± 0.5 pF. The total capacitance can be written as the sum of capacitances between every two plates:

(1) $$C=\left(N-1\right)\frac{\varepsilon_{0}A}{d}\varepsilon_{r}=C_{0}\varepsilon_{r}$$

where *N* in the total number of plates (41), *A* and *d* are the area of each plate and the separation between them respectively, $\varepsilon_{0}$ and $\varepsilon_{r}$ are, respectively, the relative permittivity of vacuum and of the medium between the plates respectively. Since the sensor will be used to detect changes in $\varepsilon_{r}$, sensitivity can be defined as in Equation (2) and is expected to provide a sensitivity value of 136.7 pF for a change in $\varepsilon_{r}$ of 1.

(2) $$S=\frac{\partial C}{\partial \varepsilon_{r}}=C_{0}$$

The sensor was dipped in the oil with terminals being applied an AC signal. The relative permittivity can be calculated from the measured $C_{0}$ and $C_{oil}$:

(3) $$\varepsilon^{{}^{\prime }}=\frac{C_{oil}}{C_{0}}$$

The dielectric losses and the conductivity can be calculated from the measured parallel resistance and measured capacitance $C_{0}$:

(4) $$\varepsilon^{{}^{\prime \prime }}=\frac{1}{C_{0}R\omega }=\frac{\sigma }{\varepsilon_{0}\omega }$$

The capacitance *C* and the resistance *R* are calculated from the measured values as in the following equation:

(5) $$Y\left(\omega \right)=\frac{1}{Z(\omega )}=G+j\omega C=\omega C_{0}\varepsilon^{{}^{\prime \prime }}+j\omega C_{0}\varepsilon^{{}^{\prime }}$$

where *G* is the conductance (G=1/R) and *C* is the capacitance.

**Figure 4:**
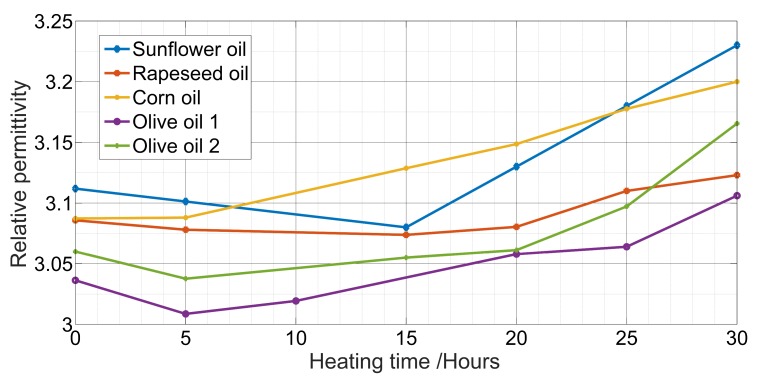
Measured relative permittivity vs. the heating time.

In the frequency range 10 kHz to 1 MHz, the capacitance was constant with a minor change in the range of 10 fF. In this range of frequency, the relative permittivity was independent of the frequency which conforms to [16]. The measured value of the relative permittivity of different oils was in the range of 3 and 3.25 with a variation as small as 0.01 (Figure 4). In fact, it decreased between 0 and 5 h and then increased with the increase of the heating time. The first decrease was caused by water evaporation due to heating.

**Figure 5:**
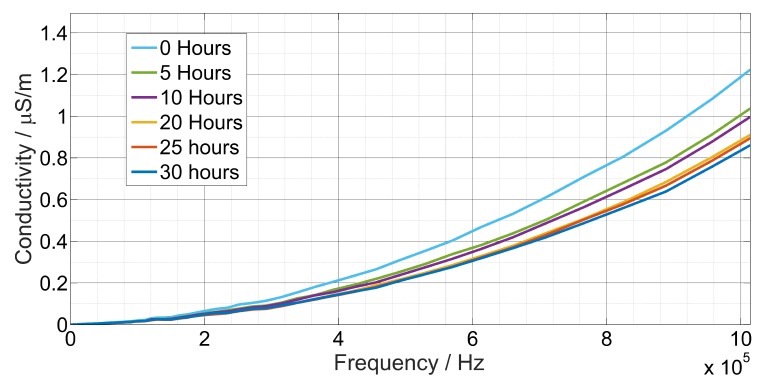
The measured conductivity function of heating time and frequency for sunflower oil.

**Figure 6:**
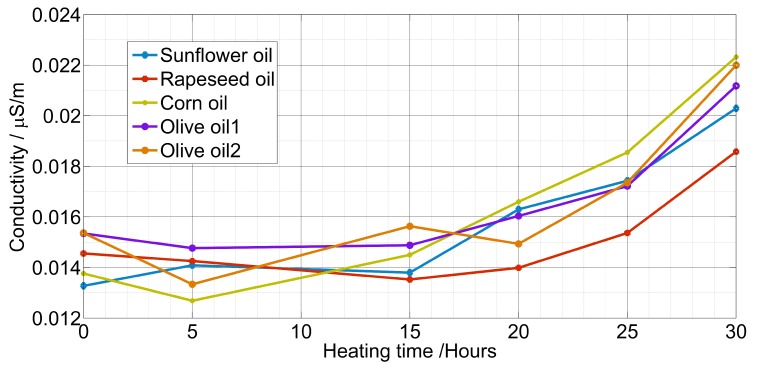
Measured conductivity at 10 kHz vs. the heating time.

The conductivity σ of the oil depended strongly on the frequency (Figure 5), it increased proportionally in the function of frequency from the range of nS/cm to the range of µS/cm. Experimental results for the conductivity as a function of heating hours are plotted in Figure 6.

A decrease in the relative permittivity and conductivity was observed for some oil samples due to the water and volatile compounds evaporation. After 15 h, both of the quantities increased. The first quantity increase was induced by the newly formed components such as free fatty acids, aldehydes, ketones, and alcohols [45]. Whereas the second quantity was due to the increase of kinetic energy thanks to the decrease of oil density allowing for a more molecules mobility [46].

### 2.3. Regression Analysis

Regression analysis was performed to correlate between the TPC with the relative permittivity and the acidity with the conductivity. The conductivity of oil was determined by the composition of its fatty acid composition. Partial least square regression analysis was made at a constant frequency of 100 kHz. The results of these analyses are shown in Table 1, and the results were evaluated with a coefficient of determination $R^{2}$.

Similarly, the relative permittivity and total polar compounds regressed by first-order polynomial equations, with results shown in Table 2.

## 3. Measurement Setup

### 3.1. Capacitive Sensor

**Figure 7:**
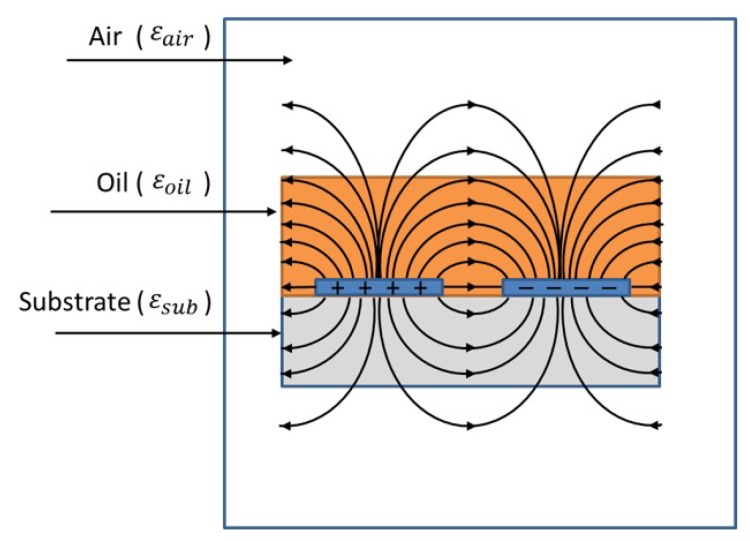
Electric field distribution between two electrode fingers of an inter-digital electrodes sensor.

An inter-digital electrodes (IDE) sensor is convenient for oil measurement since it can be easily dipped in oil and cleaned after being used. The theoretical value of the IDE capacitance was calculated from the combination of the parallel plate capacitor formula in addition to the stray field in elliptic form (Figure 7). In addition to that, three mediums exist around the electrodes: The substrate with a relative permittivity $\varepsilon_{sub}$, the medium under investigation (oil in our case) with a relative permittivity $\varepsilon_{oil}$, and the air with $\varepsilon_{air}=1$. Since $\varepsilon_{sub}$, $\varepsilon_{oil}$ and $\varepsilon_{air}$ are different, the distribution of the electrical field in these mediums is different.

**Figure 8:**
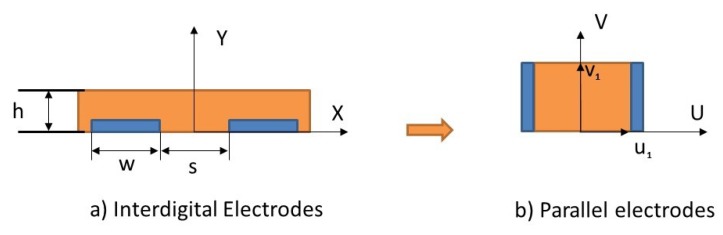
Representation of two fingers structure in the (x, y) plane (**a**), and after conformal mapping in the (u, v) plane (**b**).

The capacitance can be determined using Laplace’s equation when the potential distribution for every point in the intermediate space is known. However, practically this is deemed complicated. Alternative methods such as the analytical evaluation of inter-digital electrodes have been established since 1977 [47]. The proposed model is based on the conformal mapping (CM) technique to evaluate the capacitance of an IDE sensor, which transforms the field lines into on a vertical plane (u, v) (Figure 8). The capacitance of two electrodes can then be written as:

(6) $$C=\frac{\varepsilon_{0}\varepsilon_{r}Lv_{1}}{2u_{1}}$$

where *L* is the length of the electrodes (not shown in Figure 8), $v_{1}$ is the width of an electrode, and $u_{1}$ is half of the distance between two electrodes in the (u, v) plane. The main two used functions in the CM transformation were the Jacobian elliptic function and the elliptic integral function.

Based on the Wei model, Olthuis et al. [48] proposed an analytical expression of the capacitance *C*, the resistance *R*, and the cell constant κ of an IDE sensor. If the sensor is composed by *N* electrodes and has a length *L*, the total capacitance is:

(7) $$C=\left(N-1\right)L\left(\varepsilon_{0}\frac{\left(\varepsilon_{r}+\varepsilon_{sub}\right)}{2}\frac{K[\sqrt{\left(1-k^{2}\right)}]}{K[k]}\right)$$

where $\varepsilon_{oil}$ and $\varepsilon_{sub}$ are the relative permittivity of oil and substrate respectively, the function K[k] is the complete elliptic integral of the first kind, and *k* is the modulus of the function [48,49]. For two conductors separated by a dielectric medium with non-zero conductivity σ, the following expression can be derived using Ohm’s law and Maxwell’s equations:

(8) $$RC=\frac{\varepsilon_{0}\left(\varepsilon_{oil}+\varepsilon_{sub}\right)}{\sigma }$$

Therefore if the capacitance *C* and the resistance *R* (in Ω) of the sensor are measured, the relative permittivity and the conductivity σ (in Sm^−1^) can be directly obtained. The cell constant of the sensor κ (in m^−1^) can be expressed in function of the resistivity ρ (in Ωm), the measured resistance *R*, the conductivity, the measured capacitance *C*, and the relative permittivity:

(9) $$\kappa =\frac{R}{\rho }=R\sigma =\frac{\varepsilon_{0}\left(\varepsilon_{r}+\varepsilon_{sub}\right)}{C}$$

The selection of the sensor geometry is very important in assuring an accurate measurement. In fact, for inter digital electrodes, the resolution can be increased by increasing the surface of the electrodes and decreasing the distance between them. This makes the electric field better concentrated between the electrodes in the material under test and decreases the effects of the stray capacitances.

A standard FR4 substrate was used for the inter-digital electrodes sensor. It had 70 electrodes, the width of each electrode was 150 µm, the length was 40 mm, and the distance between the electrodes was 150 µm. The sensor geometry was approximately 21 mm × 40 mm × 1.6 mm. Theoretically, it had a capacitance of 75 pF in the air approximately. This capacitance value was the sum of the capacitance in the substrate and in the air. In fact, since FR4 had a relative permittivity $\varepsilon_{sub}$ in the range of 4.5, the contribution of the substrate in the capacitance was 4.5 times higher than the air.

In the case that the sensor was dipped in oil with a relative permittivity equal to 3, the sensor had theoretically a capacitance of 115 pF approximately. The capacitance of the sensor had an offset capacitance, the capacitance in the substrate, and a variable capacitance that depended on the relative permittivity of oil. If we consider the application where the sensor will be used to detect changes in $\varepsilon_{r}$, then we can define the resolution as:

(10) $$S=\frac{\partial C}{\partial \varepsilon_{r}}=\frac{\left(N-1\right)L\varepsilon_{0}}{2}\frac{K[\sqrt{\left(1-k^{2}\right)}]}{K[k]}$$

Theoretically, the sensor can provide a resolution of 24 pF for 1 change in the relative permittivity. By printing the electrodes from both sides, it is possible to double the resolution of the sensor. Hence, the second IDE sensor could provide 48 pF change for 1 change in relative permittivity (corresponding to 0.48 pF for 0.01 change).

The conductivity of edible oils, at frequencies lower than 1 MHz, was usually in the range of nSm^−1^. It varied also depending on the quality of the oil, the water content, the temperature, and the frequency. The relation between the cell constant of the sensor κ (in m^−1^), the resistivity ρ (in Ωm), the measured resistance R (in Ω), the conductivity σ (in Sm^−1^), the measured capacitance C, and the relative permittivity can be written as:

(11) $$\kappa =\frac{R}{\rho }=R\left(\sigma_{r}+\sigma_{sub}\right)=\frac{\varepsilon_{0}\left(\varepsilon_{r}+\varepsilon_{sub}\right)}{C}=0.5$$

The value of κ is calculated using Equation (13). If we consider that the conductivity is in the range 1 nSm^−1^ to 100 nSm^−1^ then the resistance R is in the range 500 MΩ to 5 MΩ. In order to detect 0.1 nSm^−1^ change in the conductivity a change of 500 kΩ in the resistance should be detected.

### 3.2. Measurement Circuit

The sensor model is a complex impedance with a capacitance in parallel to a resistance. In order to measure the complex impedance, an interface circuit that uses both the conversion of impedance to voltage and to phase shift techniques is proposed. Using only one technique, it is not possible to measure *C* and *R* while considering the sensor as a pure capacitor and ignoring the presence of the resistance *R* leads to a high measurement error of the capacitance *C*. The impedance to voltage technique was used to measure the gain of the impedance while the impedance to phase shift was used to measure the phase of the impedance.

**Figure 9:**
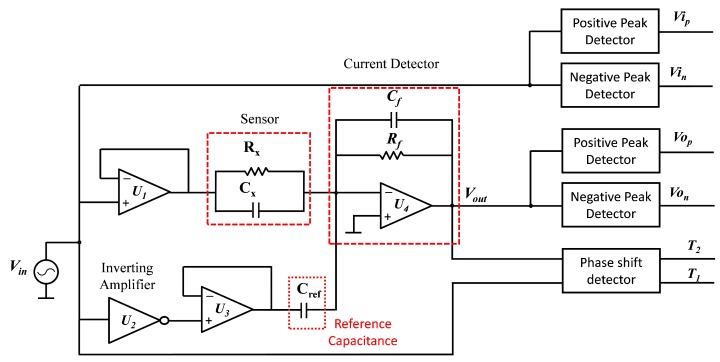
Impedance measurement circuit based on the conversion of impedance to voltage and phase shift.

**Figure 10:**
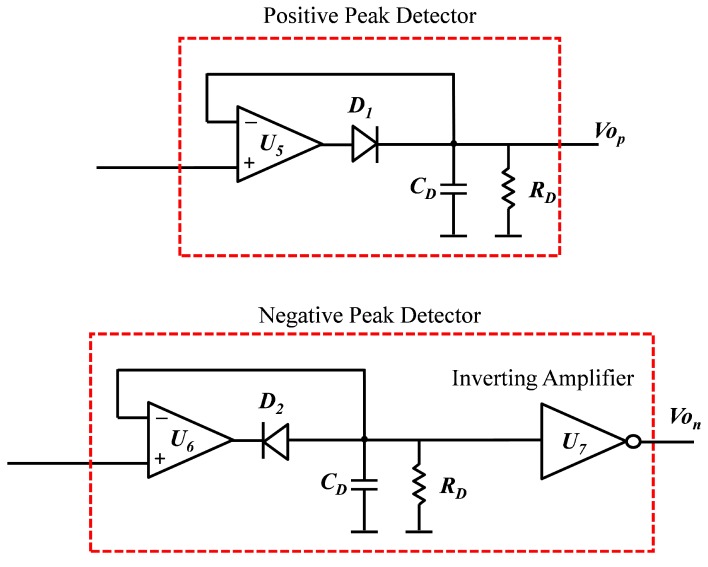
Positive and negative peak detector circuits.

The proposed measurement circuit is presented in Figure 9. The sensor and reference capacitance are excited with 180° phase shifted sinusoidal signals. The current flowing through the sensor is summed with that passing through the reference capacitance $C_{ref}$ and then converted to voltage $V_{out}$ using the current detector. The reference capacitance was used to compensate the nominal capacitance of the sensor since only a small capacitance change was to be detected. The positive and negative voltage peaks of the voltages $V_{in}$ and $V_{out}$ were detected using the positive and negative peaks detectors respectively (Figure 10). By measuring the positive and negative peaks of both input and output signals $V_{in}$ and $V_{out}$, it was possible to compensate the offset voltages as well as the variation in the amplitude and effect of the noise signals.

**Figure 11:**
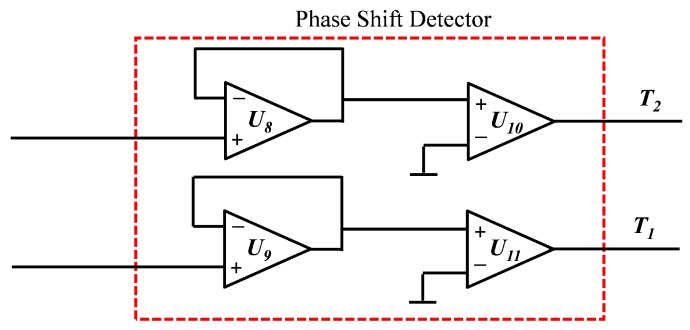
Phase shift detector circuit.

Since only the measurement of the voltage was not sufficient for calculating the impedance of the sensor, a phase shift detector was used. This circuit consisted of two comparators that compare the signals $V_{in}$ and $V_{out}$ with the ground and output a positive pulse when the signal is positive, and zero when the signal is zero or negative (Figure 11). By comparing the output signals of the comparators, the phase shift can be calculated. Nowadays micro-controllers are able to simultaneously read digital signals with a resolution of 100 ns and smaller. Simultaneously reading two digital signals is a need in our application since the phases of both signals were to be measured at the same time.

**Figure 12:**
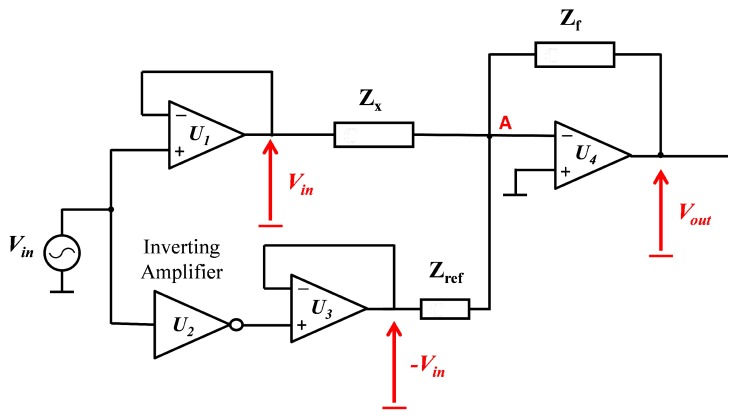
Current detector circuit including the impedance to be measured $Z_{x}$ and reference impedance $Z_{ref}$.

To better understand the working principle and behavior of the circuit, it is important to study it in the frequency domain and to study its transfer function $V_{out}/V_{in}$. The superposition theorem can be applied at node A (inverting input of the amplifier $U_{4}$) since it is considered a summer amplifier. The currents of the impedances $Z_{x}$ and $Z_{ref}$ were summed and fed to the impedance $Z_{f}$ (Figure 12).

(12) $$I_{Z_{f}}=I_{Z_{x}}+I_{Z}{}_{ref}$$

Since the non-inverting input of $U_{4}$ was connected to the ground, the inverting input was virtually grounded and the following equations could be derived:

(13) $$V_{out}=-Z_{f}\left(\frac{V_{in}}{Z_{x}}-\frac{V_{in}}{Z_{ref}}\right)$$

where:

$$Z_{x}=\frac{R_{x}}{1+j\omega C_{x}R_{x}},Z_{f}=\frac{R_{f}}{1+j\omega C_{f}R_{f}},Z_{ref}=\frac{1}{jC_{ref}\omega }$$

The transfer function T(ω) can be written as:

(14) $$\begin{array}{c} \begin{array}{cc} T(\omega ) & =\frac{V_{out}}{V_{in}}=-\frac{R_{f}}{1+j\omega C_{f}R_{f}}\left(\frac{1+j\omega C_{x}R_{x}}{R_{x}}-j\omega C_{ref}\right)\\ \; & \;=-\frac{\frac{R_{f}}{R_{x}}+j\omega R_{f}\left(C_{x}-C_{ref}\right)}{1+j\omega C_{f}R_{f}} \end{array} \end{array}$$

The Gain of T(ω) can be written as:

(15) $$\begin{array}{c} A=\left|T\left(\omega \right)\right|=\frac{\sqrt{{\left(\frac{R_{f}}{R_{x}}\right)}^{2}+{\left(R_{f}\omega \left(C_{x}-c_{ref}\right)\right)}^{2}}}{\sqrt{1+{\left(\omega C_{f}R_{f}\right)}^{2}}} \end{array}$$

The phase of T(ω) can be written as:

(16) $$\begin{array}{c} \phi =phase\left(T\left(\omega \right)\right)=tan^{-1}\left(\frac{\omega \left(\frac{C_{f}R_{f}}{R_{x}}-\left(C_{x}-C_{ref}\right)\right)}{-\frac{1}{R_{x}}-\omega^{2}R_{f}C_{f}\left(C_{x}-C_{ref}\right)}\right) \end{array}$$

It can be seen that the circuit behaved similarly to a high pass filter but with a particularity that it had a gain $A_{DC}$ in the frequencies lower than the cut-off frequency. In the general case where $C_{x}$ and $R_{x}$ were different than 0 and at constant $R_{f}$, $C_{f}$, and $C_{ref}$, the magnitude *A* and the phase ϕ depended on $C_{x}$, $R_{x}$ and the frequency. At low frequencies and particularly in DC mode, *A* and ϕ mainly depended on $R_{x}$ while at a high frequency, they mainly depended on $C_{x}$. At high frequencies where the condition ($\omega C_{f}R_{f}{)}^{2}>>1$ was met, the gain did not depend on the frequency anymore.

(17) $$A=\left|T\left(\omega \right)\right|=\frac{C_{x}-C_{ref}}{C_{f}}$$

For the input signal, the waveform generator AD9833 from analog devices was used since it provides a software programmable output frequency from DC to 12.5 MHz. The main drawback of this module was that the amplitude of the output voltage was not constant and decreased as the frequency increased, particularly for frequencies higher than 100 kHz. At 10 kHz and lower frequencies the output frequency was constant and had a 315 mV amplitude and a 330 mV DC offset voltage.

In order to cancel the offset voltage, a RC high pass filter was used. The cut-off frequency was selected experimentally to be 10 Hz in order to cancel the maximum DC offset. The signal was then amplified using a non-inverting amplifier and fed into the sensor, and inverted to get 180 phase shifted signal and fed into the reference capacitance. Two voltage followers were used to prevent the flow of current between the sensor, the reference capacitance, the inverting amplifier, and the non-inverting amplifier.

The voltages $V_{Cx}$ and $V_{Cref}$ that were applied to the sensor and reference capacitance created two 180 degrees phase shifted currents $I_{x}$ and $I_{ref}$ which flowed through the sensor impedance and reference capacitance. The sum of current was then fed to the feedback impedance $C_{f}$ and $R_{f}$ and thus converted to voltage $V_{out}$. The current $I_{x}$ contained the current flowing through the resistance $R_{x}$ and capacitance $C_{x}$. The nominal current flowing through the capacitance $C_{x}$ was compensated by the current flowing through the capacitance $C_{ref}$ and accordingly the nominal capacitance of $C_{x}$ was compensated by $C_{ref}$. This means that only the difference between these two capacitances ΔC was converted to voltage and contributed in $V_{out}$.

The positive and negative peak detectors were implemented to detect the positive and negative peaks of the signal $V_{cx}$, the voltage of the sensor, and $V_{outf}$, the output voltage of the filter. For each signal, a positive and negative peak detector was used. The negative peak detector was followed by an inverting amplifier to convert the negative voltage to a positive voltage since microcontrollers can not read negative voltages.

For the positive peak detector, when the input $V_{1}$ exceeded the voltage $V_{out1}$, the diode $D_{1}$ was forward biased and the circuit became voltage follower and the capacitor $C_{D}$ was charged. Consequently, the output voltage followed the input voltage. When $V_{1}$ dropped below $V_{out1}$, the diode became reverse biased and the capacitor $C_{D}$ was disconnected from the amplifier output and held the charge till the input voltage again had a voltage higher than $V_{out1}$. The negative peak detector worked exactly the inverse of the positive peak detector since the diode was reversely connected in comparison to the positive detector. When the input voltage $V_{2}$ was lower than the capacitance voltage, the amplifier worked as a voltage follower and the capacitance $C_{D}$ was charged. The moment $V_{2}$ became higher than the capacitor voltage, the diode disconnected the capacitor from the amplifier. The capacitor held the negative voltage until $V_{2}$ decreased lower than its voltage. An inverting amplifier was used to inverse the voltage so that it would be possible to read it with a micro-controller.

**Figure 13:**
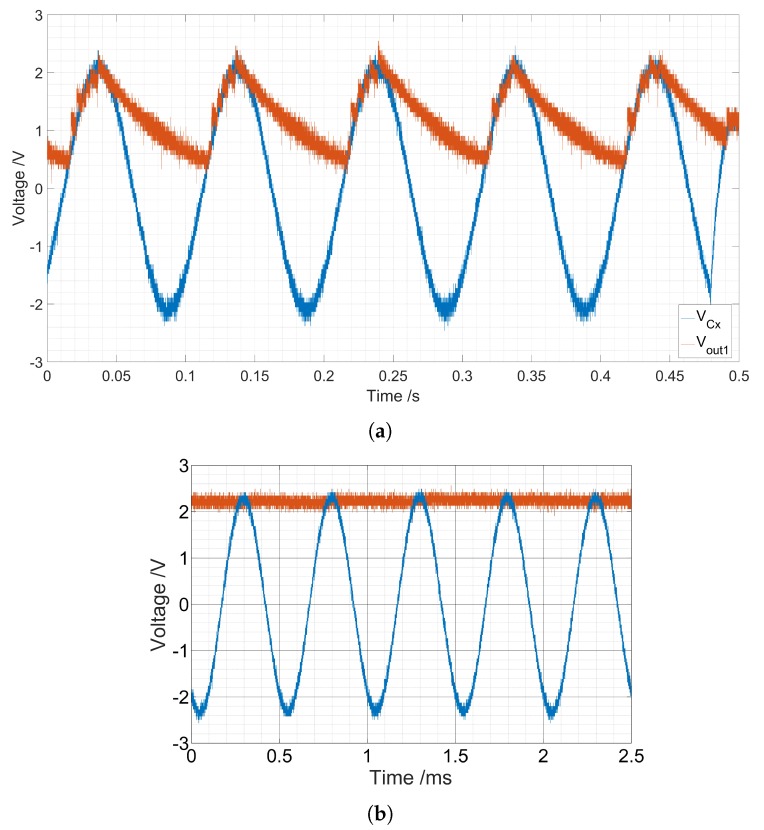
Example of the output of the peak detector at different frequencies where the discharging effect of the capacitor *C_D_* is clear. (**a**) The sensor voltage *V_CX_* and output of the positive peak detector *V_out_*_1_ at 10 Hz. (**b**) The sensor voltage *V_CX_* and output of the positive peak detector *V_out_*_1_ at 1 kHz.

Despite the used amplifiers having a high output resistance, in the GΩ range according to the datasheet, the used diode (BAT20) had a very low leakage current, a typical value of 650 nA according to the datasheet, a reverse current flowed through the diode to the amplifier and discharged the capacitance $C_{D}$. This phenomenon happens especially at low frequencies since the period was higher and the capacitance had a longer period of time to discharge (Figure 13).

Experimental results show the necessity of using a voltage follower between the signals and comparator. In fact, when the comparators were directly connected to the output of the current detector and input signals, the signal became distorted. This was caused by the high offset voltage and bias current of the comparator which was fed back to the feedback impedance of the current detector. In fact the used comparator, the AD8469, had an offset voltage of ±5 mV and bias current of ±4 µA maximum. The addition of a voltage follower between the comparators and current detector as well as the input signal prevented any current or offset voltage to be applied from the comparator on the other part of the circuit.

By comparing the two output signals of each comparator $V_{comp1}$ and $V_{comp2}$ which represent the phase of the input signal $V_{cx}$ and the current detector signal $V_{outf}$ respectively, it is possible to deduce the phase shift between both signals. Measuring the time difference between the rising or the falling edges of the first and second signal and detecting which signal makes the rise or the fall first can give information about the phase shift between the signal and thus deduce the angle of the sensor impedance. At low frequencies (100 Hz and lower), the outputs of the comparators become noisy during the transition from low to high or high to low and multi rises and falls exist in the same transition instead of only one. At low frequencies, both the voltage noise density and measurement error increase. This happened especially for the second comparator which represents the voltage of the current detector $V_{outf}$ since it had a variable amplitude that could be as low as the noise amplitude. The measurement error caused by this multi rises and falls could be reduced by averaging the first and last rises or falls of the same transition.

**Figure 14:**
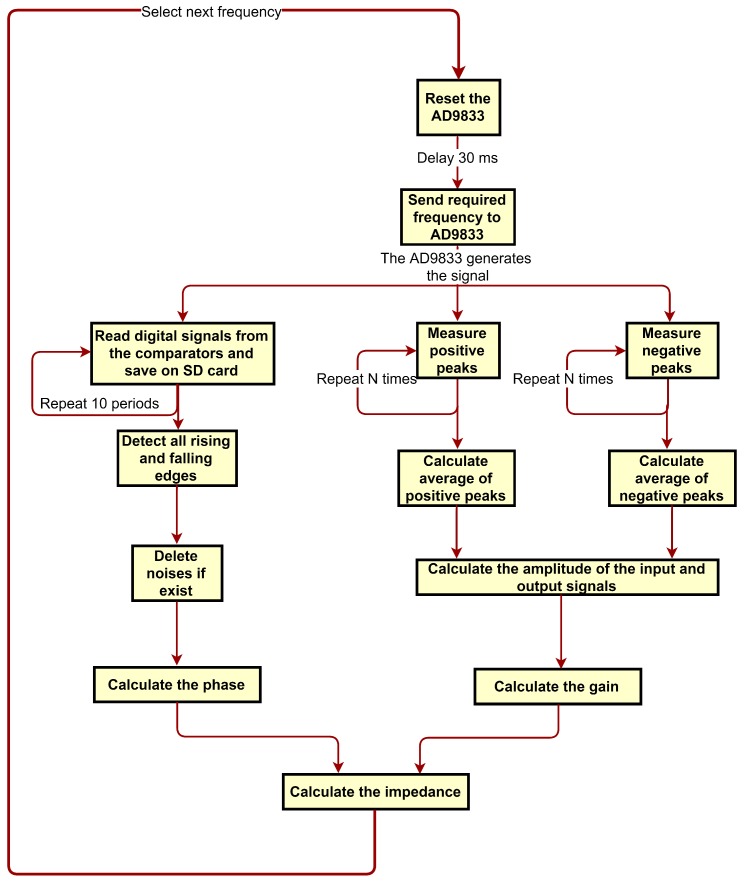
Chart flow of impedance measurement.

In order to measure the values of $C_{x}$ and $R_{x}$ accurately, the measurement should be done at different frequencies. The gain depends mainly on the capacitance at high frequencies (1 kHz and 10 kHz) and on the resistance at low frequencies (100 Hz and 10 Hz). The measurement of the phase should be done at 500 Hz and lower since this is the range where the phase had its maximum change with the change of impedance. The measurement at DC was not possible since the high pass filter at the input stage would not allow DC signals to pass to the sensor. The chart flow of the impedance measurement is explained in Figure 14.

## 4. Measurement Results

The measurement circuit was connected to the IDE sensor and the impedance of the sensor was measured at 100 Hz and 1 kHz. The impedance was measured at two different frequencies in order to calculate the complex impedance of the sensor. The values of the measured capacitance and resistance were correlated with the relative permittivity and conductivity measured in the reference measurement. It should be mentioned that the resistance and conductivity were measured at different ranges of frequency. In fact, using the impedance analyzer, it was possible to measure the conductivity only at frequencies higher than 10 kHz while the resistance was measured at 1 kHz and 100 Hz since the measurement accuracy of the measurement system decreased for frequencies higher than 1 kHz. In the case of relative permittivity and capacitance, these parameters were almost constant between 100 Hz and 1 MHz.

Many studies have been done in order to establish a coherent relation between chemical and dielectric parameters. In the researches done in [16,17,50], a linear relation between the acidity and conductivity is found with a $R^{2}$ coefficient equal to 0.999, 0.959, and 0.825 respectively. In another study [14], it was found that the acidity correlated to the relative permittivity with a $R^{2}$ coefficient equal to 0.7255. For total polar compounds (TPC), in some of the studies, TPC correlated with the conductivity with a $R^{2}$ equal to 0.9582 [51]. In other studies, TPC correlated with relative permittivity and capacitance with a $R^{2}$ equal to 0.9 [52].

The variation of relative permittivity was strongly related to the polarization phenomena. The polarization describes how a dielectric medium responses to an applied external field. The polar molecules possess a randomly orientated permanent electric dipoles in the absence of an electric field, whereas, the non-polar molecules have no permanent electric dipoles. When the dielectric material is placed in an external electric field, the dipoles become partially or fully aligned in the direction of the electrical field. Polar molecules have the geometrical arrangement in such a way that one end of the molecule has a positive electrical charge and the other side has a negative charge. In the opposite case, the molecule is non-polar. The application of an electric field rotates the permanent dipoles already in existence and induces dipoles from the non-polar molecules to have the arrangement of the polar molecules. The permittivity of the rotated polar molecules is higher than that of the induced non-polar molecules. We can conclude that if the polar molecule in oil increased, the relative permittivity also increased. For this reason, it is evident to correlate the relative permittivity with the total polar compounds in the oil. From the other side, free fatty acids which are chains of acids liberated from triglycerides were conductive mediums since they gain free electrons when liberated. For this reason, the increase of the free fatty acids could be correlated to the increase of conductivity.

The correlation of relative permittivity measured in the reference measurement and measured capacitance is shown in Table 3. As expected in a theoretical study, the measurement system provided high resolution to a small change of the relative permittivity. The measured capacitance changed linearly with the value of relative permittivity with a $R^{2}$ as high as 0.97 and 0.51 in the worst case.

**Figure 15:**
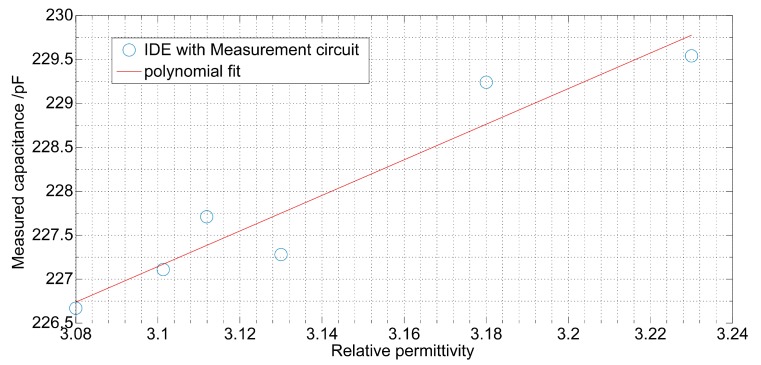
Correlation of the measured capacitance with the relative permittivity of sunflower oil measured in the reference measurement. The measurements are carried out by the measurement circuit and the IDE (inter-digital electrodes) sensor.

**Figure 16:**
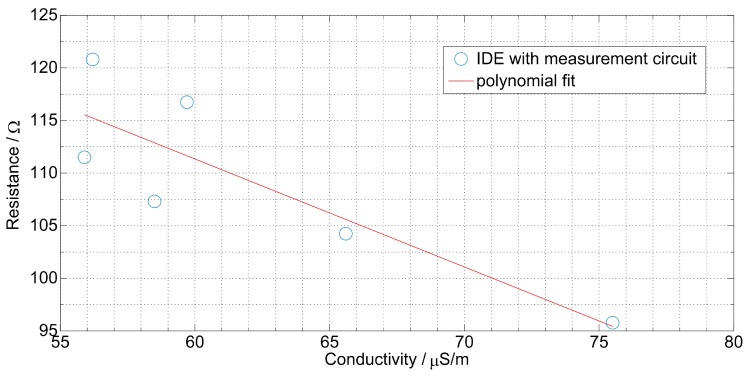
Correlation of the measured capacitance with the relative permittivity of sunflower oil measured in the reference measurement, the measurements are carried out by the measurement circuit and the IDE sensor.

The measurement of the resistance was less accurate than the measurement of the capacitance. In fact, the parallel low resistance of the substrate to the oil resistance strongly affected the measurement. Despite this effect, the relation between the conductivity and resistance was also linear with a $R^{2}$ as high as 0.91 and 0.42 in the worst case (Table 4). Figure 15 and Figure 16 show an example of the correlation for sunflower oil.

In the reference measurement, it was demonstrated that the relative permittivity and conductivity could be used to find the total polar compounds and acidity of edible oils at different heating times. The correlation done in this part served to evaluate if the measured capacitance and resistance using the measurement system could be used to calculate the relative permittivity and conductivity and thus deduce the total polar compounds and acidity. It also served to calibrate the measurement system since the measured capacitance and resistance were not in the same range as the values in the reference measurement.

## 5. Conclusions

A measurement system for frying oil quality assessment was studied and developed. The developed system was suitable for the measurement of capacitive sensor impedance where high accuracy measurement was required. The measurement procedures based on the conversion of impedance to voltage and phase shift were used for the measurement circuit, while the interdigital electrodes were selected as a capacitive sensor since they could provide high resolution for small sensor geometry.

A comparison study based on simulation between different measurement procedures of sensor impedance was done. Some of the circuits had a variable resolution while others had a limited one. Although it had a limited resolution, the bridge circuits could provide a stable and accurate measurement. This technique could be easily implemented and used since the output of the circuit was a DC voltage which could be easily measured. The circuit was immune to stray capacitances and it was possible to measure the capacitance at high frequencies where the effects of the parallel resistance were low. This technique was combined with the phase detection technique in order to measure the resistance value. Using both techniques, it was possible to measure the gain and phase of the sensor impedance at any frequency.

An interdigital electrodes sensor was used since it could provide high sensitivity for small sensor geometry, besides being easy to used in our application. The geometry of the sensor was investigated with theoretical study and simulation.

In the first investigation, a reference measurement was done. It was used to calibrate the system and consisted of chemical and dielectric measurements. The chemical ones included the acidity and total polar compounds and the dielectric included the relative permittivity and conductivity. The acidity measurement was done using the titration technique and the total polar compounds used the testo 700. The dielectric measurement was done using a parallel plate sensor and a precise impedance analyzer.

From this reference measurement, it was shown that the chemical parameters could be correlated with the dielectric parameters. The acidity was correlated with the conductivity and the total polar compounds were correlated with the relative permittivity with a $R^{2}$ as high as 0.97. This proved the potential of dielectric spectroscopy to measure the chemical properties and thus for the assessment of edible oil.

The reference measurements were then used to evaluate the performance of the measurement system. Dielectric parameters were correlated to the impedance measured using the system. The results showed an accurate measurement of capacitance where the correlation with the relative permittivity was as high as 0.97 while the measurement of the resistance was relatively less accurate with a correlation with the conductivity as high as 0.91. Compared to the reference measurement where an accurate impedance analyzer was used, the measurement using the proposed embedded solution showed its performance in the capacitance measurement and its weakness in the resistance measurement. In fact, edible oils were high ohmic mediums with low values of conductance losses which explain the high error value of the resistance measurement shown in Table 4. 

## Figures and Tables

**Table 1:** Correlation between acidity AC and conductivity σ.

Sample	Relationship	*R* ^2^
Sunflower oil	AC = 0.9948σ + 0.0005751	0.99
Rapeseed oil	AC = 0.9458σ + 0.007019	0.94
Corn oil	AC = 0.9669σ + 0.007539	0.96
Olive oil 1	AC = 0.9982σ + 0.0005035	0.99
Olive oil 2	AC = 0.9938σ + 0.00193	0.99

**Table 2:** Correlation between TPC (total polar compounds) and relative permittivity $\varepsilon_{r}$.

Sample	Relationship	*R* ^2^
Sunflower oil	TPC = 122.1$\varepsilon_{r}$ − 369	0.93
Rapeseed oil	TPC = 149.5$\varepsilon_{r}$ − 454	0.89
Corn oil	TPC = 131.9$\varepsilon_{r}$ − 398.1	0.99
Olive oil1	TPC = 96.71$\varepsilon_{r}$ − 289.6	0.82
Olive oil2	TPC = 108.4$\varepsilon_{r}$ − 327.2	0.99

**Table 3:** Correlation between relative permittivity $\varepsilon_{r}$ and the measured capacitance using the IDE sensor and the measurement circuit.

Sample	Relationship	*R* ^2^
Sunflower oil	C = 20.24$\varepsilon_{r}$ + 164.4	0.91
Rapeseed oil	C = 28.66$\varepsilon_{r}$ + 140.6	0.79
Corn oil	C = 25.04$\varepsilon_{r}$ + 151.2	0.97
Olive oil	C = 21.63$\varepsilon_{r}$ + 162.1	0.90
Olive oil	C = 14.45$\varepsilon_{r}$ + 183.8	0.51

**Table 4:** Correlation between conductivity σ and the measured resistance using the IDE sensor and the measurement circuit.

Sample	Relationship	*R* ^2^
Sunflower oil	R = −1.026σ + 172.9	0.74
Rapeseed oil	R = −5.607σ + 148.2	0.45
Corn oil	R = −0.932σ + 163.9	0.69
Olive oil	R = −1.212σ + 230.1	0.91
Olive oil	R = −1.171σ + 232.4	0.42
